# Evaluating Adjusted ssGBLUP Models for Genomic Prediction and Matrix Compatibility in South African Holstein Cattle

**DOI:** 10.3390/ani16030357

**Published:** 2026-01-23

**Authors:** Kgaogelo Stimela Mafolo, Michael D. MacNeil, Frederick W. C. Neser, Mahlako Linah Makgahlela

**Affiliations:** 1 Animal Production, Agricultural Research Council, Private Bag X2, Irene 0062, South Africammakgahlela@arc.agric.za (M.L.M.); 2 Department of Animal Science, University of the Free State, P.O. Box 339, Bloemfontein 9301, South Africa; neserfw@ufs.ac.za; 3 Delta G, Miles City, MT 59301, USA

**Keywords:** ssGBLUP, genomic prediction, Holstein, bias, accuracy

## Abstract

In populations with limited genotyping, single-step genomic best linear unbiased predictions (ssGBLUP) can produce biased or less accurate genomic predictions due to incompatibilities between genomic and pedigree relationship matrices. The study evaluated the impact of five alternative ssGBLUP models for genomic predictions of milk, fat, and protein yield production traits in South African Holstein cattle. The dataset included 696,413 milk production records and pedigrees of 541,325 animals. Production traits were 305-day lactation yields for milk, protein, and fat. Genotype data were based on the Illumina 50K chip v3, with 53,218 SNPs. A total of 1221 animals with genotypes and 41,407 SNP markers were in the final dataset. The five models used to estimate genomic estimated breeding values (GEBVs) were the single-step method (ssGBLUP), ssGBLUP accounting for inbreeding (ssGBLUP_Fx), ssGBLUP with unknown parent groups (ssGBLUP_upg), and two ssGBLUP models with blending, tuning, and scaling parameters set to optimum values in constructing the inverse of the unified relationship matrix (ssGBLUP_adjusted). Realized prediction accuracies were highest for ssGBLUP_adjusted models (6–7% improvements compared to ssGBLUP). Accuracy of GEBVs for milk, protein, and fat yields ranged from 0.23, 0.29, and 0.30 for both ssGBLUP and ssGBLUP_Fx, 0.26, 0.32, and 0.34 for ssGBLUP_upg, and 0.29, 0.35, and 0.37 for ssGBLUP_adjusted models, respectively. Corresponding bias, expressed as regression coefficients, ranged from 0.30, 0.31, and 0.36 for ssGBLUP; 0.31, 0.32, and 0.37 for ssGBLUP_Fx; 0.41, 0.44, and 0.49 for ssGBLUP_upg; and 0.44, 0.47, and 0.53 for ssGBLUP_adjusted models, respectively. The improved accuracy and reduced bias observed with the ssGBLUP_adjusted underscores the importance of optimizing the blending of pedigree- and genome-based relationships to achieve more reliable GEBVs, thereby improving selection decisions in Holstein dairy cattle.

## Simple Summary

A single-step genomic best linear unbiased prediction (ssGBLUP) model can produce biased or less accurate genomic predictions due to incompatibilities between genomic and pedigree information, especially in populations with limited genotypes. This study evaluated the impact of five ssGBLUP models for estimating genomic estimated breeding values (GEBVs) for milk, fat, and protein yields in South African Holstein cattle. The models included the standard ssGBLUP, the ssGBLUP accounting for inbreeding and unknown parent groups, and two adjusted ssGBLUP models incorporating blending and scaling, with and without tuning. The results showed that the adjusted models consistently produced more accurate and less biased GEBVs than the standard model. Therefore, these findings demonstrate that optimizing the integration of genomic and pedigree data can substantially enhance the reliability of genetic predictions and support more effective selection decisions, contributing to faster genetic progress in South African Holstein populations.

## 1. Introduction

Genomic prediction involves integrating pedigree and animal performance data with single-nucleotide polymorphism (SNP) markers to estimate genomic estimated breeding values (GEBVs). It has contributed to major advances in the prediction of breeding values over the past two decades, thereby supporting progress in animal breeding [1,2,3]. The use of genotypes accelerates genetic progress by improving the accuracy of prediction, which, at least in some cases, allows a reduction in the generation interval [2,4,5]. Hence, the accelerated genetic gains have been observed for several species, including cattle [6,7,8], pigs [9,10,11], chickens [12,13], goats [14,15,16], and sheep [17,18,19].

With the genomic prediction models among the critical factors affecting the accuracy of genomic predictions [20,21], the single-step genomic best linear unbiased predictions (ssGBLUP) model unifying the pedigree, genotypes, and phenotypes into a single evaluation has been widely adopted [22,23,24]. Consequently, this model uses the inverse of the unified relationship matrix (**H^−1^**), which incorporates the pedigree relationship matrix (**A**), the genomic relationship matrix (**G**) [22,23,24]. The ssGBLUP model has several advantages, such as the simultaneous evaluation of genotyped and non-genotyped animals, improved connectedness between pedigree and genomic information, and reduced bias arising from selective genotyping [23,25,26]. Therefore, ssGBLUP is important especially in populations with limited numbers of genotyped animals, which are associated with low prediction accuracy and bias [23,27]. In addition, ssGBLUP avoids the use of pseudo-phenotypes due to the direct incorporation of raw phenotypic records, which simplifies implementation while maintaining robustness for predominantly polygenic traits such as milk production [24,28,29].

Meanwhile, some challenges remain for ssGBLUP evaluations, particularly related to differences in level and scale between the **A** and **G** matrices. These discrepancies, often influenced by factors such as incomplete pedigree records, population structure, and the use of allele frequencies derived from the current population rather than the base population, can influence bias in GEBVs [24,30]. In addition, differences in scaling between **G** and the **A** matrices, as well as genotyping errors and selective genotyping, can contribute to inflation or deflation of GEBVs [31,32,33]. Therefore, the ssGBLUP model can further be improved by modifying the **H^−1^** matrix through blending, tuning, and scaling [24,30,33,34,35,36,37] as well as by accounting for unknown parent groups (UPG) and inbreeding [32,38,39,40,41,42,43]. However, these adjustments have been applied individually in previous studies [33,37,39,44], and limited work has assessed their combined effect when implemented simultaneously within a single ssGBLUP evaluation. The combined effects of these adjustments refer to their joint influence on the accuracy and stability of GEBVs when implemented simultaneously in a single ssGBLUP evaluation

Mafolo et al. [44] evaluated the accuracy of GEBVs predicted using ssGBLUP with standard blending, tuning, and scaling parameters, and compared these to alternative parameter configurations applied individually to the **H^−1^** matrix for milk production traits. However, conclusions regarding matrix compatibility were inferred from prediction accuracy rather than directly quantified, leaving the actual influence of combined adjustments on **G** and **A** matrices alignment unexplored. Previously, the compatibility between **G** and **A** was assessed statistically using descriptive statistics, regression coefficients, and their correlations [45]. This limitation is particularly important in developing countries, where reference populations are often small, female-biased, or genetically suboptimal, because relying solely on prediction accuracy may not reveal how well the **G** and **A** matrices actually align [46,47,48]. Therefore, understanding how combined adjustments influence matrix compatibility is critical to improving prediction accuracy and reducing bias in numerically small populations. While adjusting the **H^−1^** matrix is necessary, the predicted GEBVs must also be accurate and less biased, because poor calibration may produce inflated or deflated values [49,50,51]. Therefore, the objective of this study was to evaluate the impact of an adjusted ssGBLUP model on genomic predictions for milk production traits in South African Holstein cattle by assessing prediction accuracy, bias, and relationship matrix compatibility.

## 2. Materials and Methods

### 2.1. Data Sources and Editing

This study is a follow-up to Mafolo et al. [44] and uses the same pedigree and phenotypic datasets as described therein. Only records for milk, protein, and fat yield from the first three lactations, recorded between 1989 and 2016, were included. Pedigree and phenotypic data editing followed the procedures described in Mafolo et al. [44]. The final dataset, as originally compiled by Mafolo et al. [44], is summarized in Table 1. Contemporary groups were defined as herd-year-season (HYS) of calving, with calving seasons designated as summer (October to March) and winter (April to September). Only contemporary groups with at least five animals and progeny of at least two sires were included. Trait means were identical to those reported by Mafolo et al. [44]: 7940.1 kg, 290.23 kg, and 242.54 kg for milk, protein, and fat yield, respectively.

Genotyping followed the protocol described by Mafolo et al. [44], with no additional genotyping performed in the present study. Only animals with EBVs reliability ≥ 60% were selected when genotyping was conducted for the Dairy Genomic Program (DGP) previously characterized by Visser et al. [52], resulting in a study population of 1473 Holstein cattle. Genotyping was performed using the Illumina 50K SNP chip v3 (Illumina Inc., San Diego, CA, USA), covering 53,218 markers. Quality control was conducted using PLINK v1.07 [53], removing markers with minor allele frequency < 0.05, genotyping call rate < 0.90, or significant deviation from Hardy–Weinberg equilibrium (*p* < 0.0001). Animals with SNP call rates below 0.90 were also removed. The final filtered genotypic dataset, identical to that used by Mafolo et al. [44], is presented in Table 1.

### 2.2. Statistical Analysis

Estimated breeding values (EBVs) for milk, protein, and fat yields were predicted using a single-trait best linear unbiased prediction (BLUP) repeatability model:

**y** = **X**b + **Z**a + **W**pe + e (1)

where y is the vector of observations for the traits; **X**, **Z** and **W** are known incidence matrices for records of fixed, random, and permanent environmental effects, respectively; b is the vector of fixed effects (HYS, age at calving, parity); a is the vector of additive genetic effects, assumed to be normally distributed N(0, **A**$\sigma_{a}^{2}$), where **A** is the additive genetic relationship matrix and $\sigma_{a}^{2}$ as the additive genetic variance; pe is the vector of permanent environmental effects (N(0, **I**$\sigma_{pe}^{2}$)); and e is the vector of residual effects (N(0, **I**$\sigma_{e}^{2}$)), wherein **I** represent identity matrices, and $\sigma_{pe}^{2}$ and $\sigma_{e}^{2}$ represent the variance of permanent environmental effects and residual, respectively.

#### 2.2.1. Single-Step Genomic Best Linear Unbiased Prediction

Five alternative ssGBLUP models were used to predict GEBVs, where **A^−1^** from the BLUP model was replaced by **H^−1^** [23,54]. The matrix **H^−1^** combines the **A** and **G** matrices as shown below:

(2) $$H^{-1}\;=\;A^{-1}\;+\;\left[\begin{array}{cc} 0 & 0\\ 0 & \tau G^{-1}{-\omega A}_{22}^{-1} \end{array}\right]$$

where all the animals in the pedigree are represented in **A**^−1^, whereas **G**^−1^ represents the inverse of the genomic relationship matrix based on VanRaden [55]. The matrix $A_{22}^{-1}$ represents the partition of the **A^−1^** matrix for the genotyped animals. Tuning was applied following Chen et al. [34], where the average of the diagonal elements of the **G** matrix equals that of the **A** matrix, and the average of the off-diagonal elements of both matrices is also equal. The scaling factors of 1 are applied to both **A** and **G** matrices (***τ*** = ***ω*** = 1). Blending is applied using the formula: **G** = (1 − β)**G** + β**A**_22_, where β represents the fraction of residual polygenic variance that is unaccounted for by **G** and is β= 0.05 as a standard in BLUPF90 [24]. Comparisons among the five ssGBLUP models were based on systematic modifications of the **H^−1^** matrix, as summarized in Table 2.

Model 1 represents the standard ssGBLUP without modifications (Equation (2)). Model 2 incorporated inbreeding in the **A^−1^** matrix [32,56]. Model 3 accounted for the UPG in the construction of **A^−1^** [57,58]. Models 4 and 5 applied optimized adjustments and incorporated these combined adjustments within a single ssGBLUP model. These adjustments included accounting for inbreeding and UPG, and modifications to the blending, scaling, and tuning of the **H^−1^** matrix. In this context, combined adjustments refer to the concurrent application of multiple ssGBLUP modifications to assess their overall influence on prediction accuracy and bias, as well as the alignment between the **G** and **A** matrices. Model 4 implemented all combined adjustments without tuning, allowing evaluation of their effect in the absence of explicit matrix alignment, whereas Model 5 applied tuning to align the **G** and **A** matrices, providing a fully optimized configuration. These configurations are summarized in Table 2. The tuning parameters β = 0.20 and ω = 0.60 are adopted from previous work [44]. However, given their importance for matrix compatibility and prediction performance, additional justification is warranted. Variance components for all models were estimated using the average information restricted maximum likelihood method (Supplementary Table S1), implemented through AIREMLF90 v1.149.

#### 2.2.2. Assessment of Prediction Accuracy

The EBVs and GEBVs were predicted using BLUPF90 v 1.63 [59] as described in our earlier study [44]. The BLUP model was used to predict EBVs from the full dataset, both with genotyped and un-genotyped animals. Subsequently, the ssGBLUP models were used to predict GEBVs from a dataset that excluded the phenotypes of 390 animals with genotypes in the analysis [44]. These animals were used as a validation population to evaluate the accuracy and bias of the ssGBLUP models.

Prediction accuracy was assessed as realized prediction accuracy, calculated as the Pearson correlation between GEBVs from the reduced dataset and EBVs from the full dataset for validation animals. This approach evaluates the ability of the models to predict breeding values in the absence of phenotypic records, while retaining phenotypic and genomic information from related animals in the population.

#### 2.2.3. Assessment of Prediction Bias

Following Mäntysaari et al. [49], bias and the inflation or deflation of genomic predictions were assessed for all the ssGBLUP models using the regression model:

Y = b_0_ + b_1_X_BV_ + e (3)

where Y represents the EBVs from the full dataset, b_0_ is the intercept, b_1_ is the linear regression coefficient, and X_BV_ represents the GEBVs derived from the reduced dataset, with **e** indicating the residual. The assessment of bias in genomic predictions involved evaluating the regression of EBVs on GEBVs [41,60]. The estimated regression slope reflects the dispersion of GEBVs relative to EBVs, with an expected value of 1 indicating unbiased predictions. Values less than 1 indicate inflation of GEBVs, whereas values greater than 1 indicate deflation. Regression coefficients were leveraged to assess bias by comparing EBVs derived from full datasets with those from reduced datasets, focusing on how adjustments in the ssGBLUP model impacted these relationships. The methodological rigor guiding the present approach to evaluating bias is drawn from previous studies [61,62].

#### 2.2.4. Analysis of Inflation or Deflation

Fat yield was selected as the trait of interest to evaluate the impact of adjustments on prediction accuracy and bias. The analysis examined how adjustments affected the overestimation and underestimation of GEBVs relative to EBVs. The comparison involved three groups: (1) all validation animals used in the analysis (*n* = 390); (2) validation animals with overestimated GEBVs, where GEBVs were greater than EBVs from the full model (51 in ssGBLUP and 52 in ssGBLUP_adjusted0); and (3) validation animals with underestimated GEBVs, where GEBVs were lower than EBVs from the full model (339 in ssGBLUP and 338 in ssGBLUP_adjusted0). Additionally, descriptive statistics of EBVs and GEBVs were compared across these groups to evaluate the effectiveness of the adjustments.

### 2.3. Compatibility Statistical Analysis

The **G** and **A**_22_ matrices were compared to assess their compatibility in different ssGBLUP models. The BLUPF90 software v 1.63 suite was used to generate descriptive statistics of the **G** and **A**_22_ relationship matrices (mean, minimum, and maximum), regression coefficients of **G** on **A**_22_ (b_0_ and b_1_), and correlation between **G** and **A**_22_ [45]. These statistics provided a quantitative basis for evaluating the compatibility between genomic- and pedigree-based relationship matrices prior to genomic prediction.

## 3. Results

### 3.1. Prediction Accuracy of Different ssGBLUP Models

**Figure 1:**
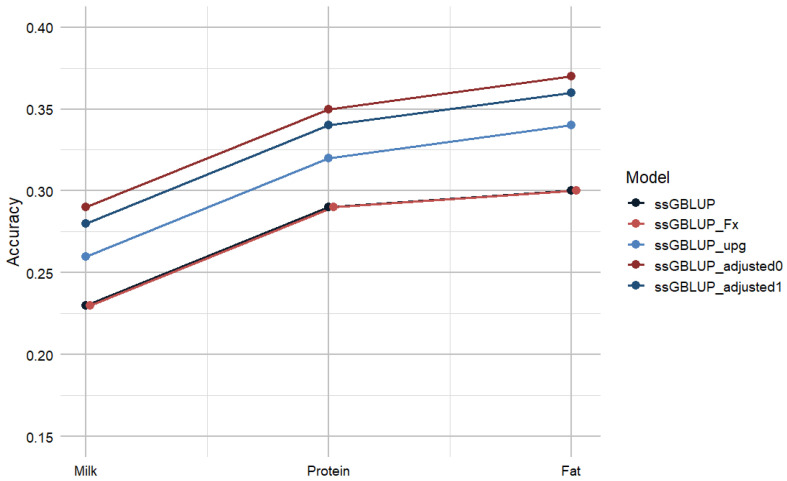
Prediction accuracy for milk, protein, and fat using different ssGBLUP models. ssGBLUP—Single-step Genomic BLUP; ssGBLUP_Fx—ssGBLUP with inbreeding; ssGBLUP_upg—ssGBLUP with unknown parent groups; ssGBLUP_adjusted0—adjusted ssGBLUP without tuning; ssGBLUP_adjusted1—adjusted ssGBLUP with tuning.

Prediction accuracy shows differences between the five ssGBLUP models in the GEBVs for milk, protein, and fat yields (Figure 1). The standard ssGBLUP model reproduced the results reported by Mafolo et al. [44], with accuracies of 0.23 for milk, 0.29 for protein, and 0.30 for fat, which were similar to the ssGBLUP_Fx model. Thus, accounting for inbreeding resulted in no improvement in the prediction of GEBVs. However, the ssGBLUP_upg model improved the estimates of accuracy to 0.26 for milk, 0.32 for protein, and 0.34 for fat, reflecting a 3% to 4% improvement relative to the ssGBLUP model. Lastly, the ssGBLUP_adjusted0 model produced the highest estimates of accuracy of 0.29 for milk, 0.35 for protein, and 0.37 for fat. Therefore, the ssGBLUP_adjusted0 model produced a 6% to 7% improvement in accuracy of the GEBVs compared to the standard ssGBLUP model and a 3% improvement in accuracy relative to the ssGBLUP_upg model. These results highlight the collective effect of combining different parameters to remodel the inverse of the **H^−1^** in ssGBLUP.

### 3.2. Bias of Genomic Predictions for Different ssGBLUP Models

**Figure 2:**
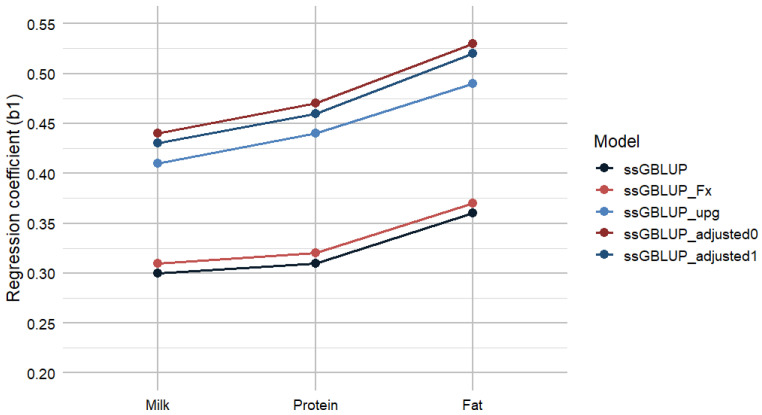
Prediction bias for milk, protein, and fat using different ssGBLUP models. ssGBLUP—Single-step Genomic BLUP; ssGBLUP_Fx—ssGBLUP with inbreeding; ssGBLUP_upg—ssGBLUP with unknown parent groups; ssGBLUP_adjusted0—adjusted ssGBLUP without tuning; ssGBLUP_adjusted1—adjusted ssGBLUP with tuning.

Regression coefficients from the regression of EBVs on GEBVs indicate bias produced by the four ssGBLUP models for milk, protein, and fat yields (Figure 2). The inclusion of inbreeding in ssGBLUP_Fx reduced bias by only 1% for all the traits. Notably, substantial reductions in bias were observed in the ssGBLUP_upg model. The ssGBLUP_adjusted models reduced estimated bias by 14–17% from standard ssGBLUP and 3–6% from the ssGBLUP_upg models. Additional details of the regression analyses, including intercepts (b_0_), coefficients of determination (R^2^), and confidence intervals, which demonstrate the enhanced fit and robustness of the ssGBLUP_adjusted0 model relative to the standard approach, are presented in Supplementary Table S2.

### 3.3. Regression Analysis for GEBVs Inflation and Deflation in Fat Yield

**Figure 3:**
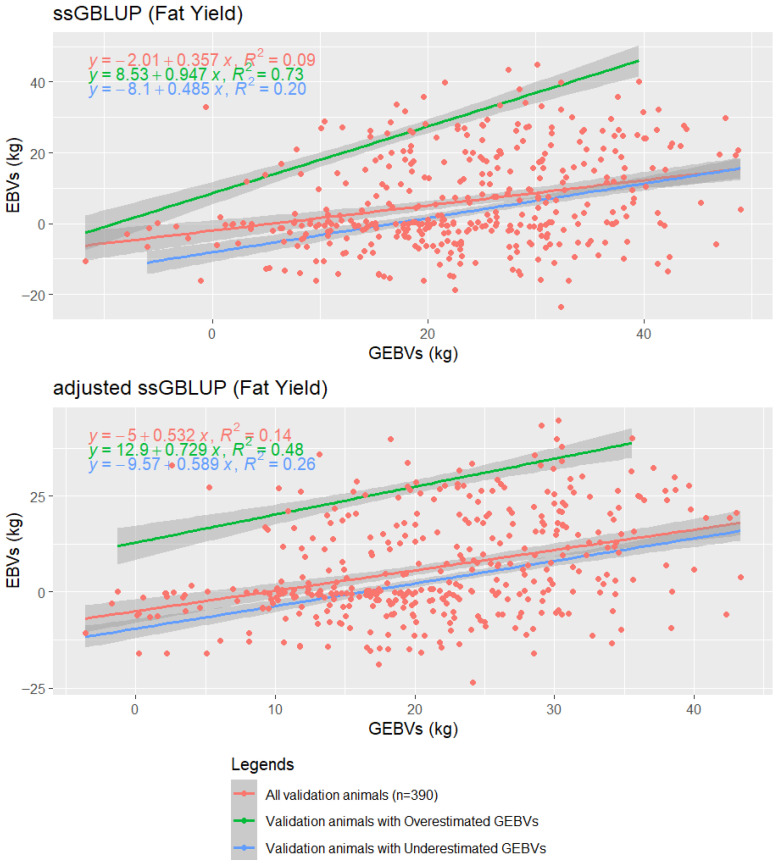
Regression analysis of fat yield in Holstein cattle, assessing prediction bias between the single-step genomic best linear unbiased prediction (ssGBLUP) and ssGBLUP_adjusted0. Estimated breeding values (EBVs) and genomic estimated breeding values (GEBVs) are expressed in kilograms (kg). Points: all 390 validation animals are shown in orange. Regression lines: orange line shows regression for all validation animals, green line shows regression for animals with overestimated GEBVs (GEBVs > EBVs; 51 for ssGBLUP, 52 for ssGBLUP_adjusted0), and blue line shows regression for animals with underestimated GEBVs (GEBVs < EBVs; 339 for ssGBLUP, 338 for ssGBLUP_adjusted0).

Figure 3 presents the regression analysis of fat yield for all 390 validation animals, comparing ssGBLUP and ssGBLUP_adjusted0 predictions. The initial comparison includes all validation animals (orange points), followed by a stratification based on prediction direction. Animals with GEBVs exceeding EBVs are classified as overestimated (green), while those with lower GEBVs than EBVs are classified as underestimated (blue). This grouping allows a clearer assessment of prediction bias patterns between the two models.

For the underestimated group, the ssGBLUP_adjusted0 showed an increased slope of 0.589 compared to 0.485 for the standard ssGBLUP, indicating a stronger relationship between GEBVs and EBVs. The R-squared also improved from 0.20 to 0.26, meaning that the GEBVs from the adjusted model explained 26% of the variation in EBVs, compared to 20% with the standard model. In contrast, for the overestimated group, the slope decreased from 0.95 in ssGBLUP to 0.73 in the adjusted model, and the R-squared dropped from 0.73 to 0.48, suggesting that ssGBLUP_adjusted0 successfully mitigated GEBVs inflation, though with a modest trade-off in predictive strength. When analyzing all validation animals, the ssGBLUP_adjusted0 slightly outperformed the ssGBLUP with a slope of 0.53 (vs. 0.36).

### 3.4. Relationship Matrix Statistics and Compatibility Statistics

Table 3 shows that the relationship matrices of the diagonal elements were close to unity across all models (0.998–1.012), indicating appropriate scaling of genetic variances. The off-diagonal elements had very small means (0.002–0.011), reflecting a population with minimal relatedness. The unadjusted **G** matrix exhibited a wider range for both diagonals and off-diagonals compared to **A**_22_ and the adjusted models (ssGBLUP_adjusted0 and ssGBLUP_adjusted1), whereas the adjustments moderated extreme values without altering the overall scale.

Correlations between genomic and pedigree relationships were consistently higher in the adjusted ssGBLUP models (0.64–0.71) than in the standard ssGBLUP (0.54–0.62) as shown in Table 4. The regression coefficients (b_1_) were closer to unity, and the intercepts (b_0_) were near zero, indicating improved agreement and minimal bias between **G** and **A**. Overall, these results suggest that the adjustments applied in the adjusted ssGBLUP models improved the consistency and reliability of genomic relationships relative to pedigree information.

## 4. Discussion

Evaluating different genomic prediction models is essential for improving accuracy and reducing bias, as their performance depends on the genetic characteristics of the trait [47,63]. This study assessed the accuracy and bias of genomic predictions using ssGBLUP and its adjusted models for milk production traits in South African Holstein cattle. Overall, all the prediction models resulted in low prediction accuracy and substantial bias. The prediction accuracy and bias are influenced by factors such as trait heritability, size and structure of the reference population, and genetic connectedness [20]. In this study, the major contributor to this low prediction accuracy and increased bias is possibly the limited size of the reference population and the structure through possible pre-selection and preferential treatment. Consequently, additive genetic variance and heritability had a slight increase under the adjusted models (Supplementary Table S1), indicating that scaling, blending, and UPG incorporation strengthened the genetic signal captured by the genomic models [39,64].

Biologically, the slight increase in heritability estimates observed under the adjusted ssGBLUP models suggests improved recovery of additive genetic variance rather than a true change in the genetic architecture of the traits [65]. By correcting scale discrepancies between **A** and **G** matrices, the adjusted models more accurately attribute phenotypic variation to inherited genetic effects, thereby reducing noise associated with population structure and selective genotyping [66]. However, evidence directly comparing heritability estimates across alternative ssGBLUP adjustments remains scarce and is largely limited to isolated evaluations of genomic scaling parameters in pig populations [39]; therefore, the present study contributes to this limited body of evidence.

The low prediction accuracies and substantial bias observed across all the ssGBLUP models can partly be explained by preselection within the genotyped reference population used in this study. This study used genotypes of cows with EBVs reliability of at least 60% as part of the selection criteria for the DGP [52]. Possibly, the genotype set was mainly of medium to high reliability animals [67]. Therefore, this could lead to preselection that reduces genetic variance, inflates relationships, and introduces bias into GEBVs [68]. Previous studies have shown that genomic preselection of young sires or top-performing cows systematically reduces accuracy, which requires the inclusion of a broader spectrum of animals to improve accuracy [69,70]. The sharp decline in correlations observed here when 390 cows were excluded from the reference population (from 0.87 to 0.26 for milk yield) underscores the sensitivity of predictions to selective genotyping [44]. In addition, preferential treatment possibly compounded this effect, as it is known to cause inflation in EBVs when such animals dominate the reference [71]. Preferential treatment occurs when certain high-performing or elite animals are managed or recorded under better conditions than the rest of the herd. This makes their records unrepresentative of the population and can inflate their EBVs when they dominate the reference group [71].

The persistence of bias and low prediction accuracy observed in this study could also be influenced by the population structure of South African Holstein cattle, which is characterized by extensive use of imported semen. The South African Holstein cattle population is shaped by high levels of imported semen, mainly from the United States of America, Canada, and Europe [52]. Despite leading to the broadening of genetic inputs, it can also narrow the effective genetic base when progeny from a few influential sires dominate, which ultimately contributes to the risk of bias [52]. Genomic relatedness analyses reported by Visser et al. [52] showed that a handful of internationally sourced artificial insemination sires strongly influence the population structure, while herds sampled more widely across South Africa displayed greater genetic diversity and heterozygosity. Therefore, this highlights the importance of ensuring adequate relatedness between reference and candidate animals to achieve reliable GEBVs and informs future genotyping strategies [72].

The improvements in prediction accuracy and bias observed for the adjusted ssGBLUP models are supported by compatibility statistics comparing the standard ssGBLUP with the adjusted implementations (Table 4). There was an improvement in the alignment between **G** and **A** matrices and the stabilizing of the **G** matrix, resulting in the ssGBLUP_adjusted0 (without tuning) more accurately capturing genetic relationships among genotyped and non-genotyped animals. These findings are consistent with Londoño-Gil et al. [45], who reported improved compatibility between **G** and **A**_22_ in multi-breed populations following similar adjustments. Despite the increase not being marginal, the results from compatibility and variance components agree with one another. Therefore, the adjustments made on the **H^−1^** reduce inflation, ensure proportional scaling, and increase the reliability of GEBVs, which likely describe the improved prediction accuracy and lowering of bias compared to the standard ssGBLUP. Similarly, Londoño-Gil et al. [45] reported improved compatibility between **G** and **A**_22_ in multi-breed populations due to adjustments. Therefore, the present study shows that optimized scaling, blending, and incorporation of UPG improved correlations and regression slopes between **G** and **A**_22_. This proves that adjustments reduced scale discrepancies and enhanced alignment of **G** and **A** relationships. In addition, alternative tuning methods provide additional theoretical support for reducing bias by improving matrix compatibility [73].

Tuning is mostly applied to align **G** with **A**_22_, either by adjusting the means of diagonal and off-diagonal elements or using allele-frequency-based methods while ensuring compatibility across matrices [33,34,64,74]. While tuning often improves prediction accuracy and reduces bias, the present study shows that its impact can be modest, particularly when scaling, blending, and UPG adjustments are already included. Interestingly, ssGBLUP_adjusted0 slightly outperformed ssGBLUP_adjusted1 in stabilizing GEBVs and reducing over-dispersion, despite small differences. These results are in line with previous research [44,75], showing that excluding tuning may provide comparable or slightly better results under specific population structures. However, the exclusion of tuning is associated with the potential of inflation and bias [74], and therefore, careful consideration is necessary during **H^−1^** adjustments based on specific populations. In addition, it should be noted that the relative performance of tuned and untuned ssGBLUP models can vary depending on factors such as the size of the reference population, genotyping density, and the history of the selection populations [44,74,75]. Therefore, the modest effects observed in this study may not directly generalize to other populations with different structures or breeding schemes.

The most notable gains in this study were achieved with ssGBLUP_adjusted0, which improved prediction accuracy by 6–7% compared to the standard ssGBLUP from Mafolo et al. [44] and reduced bias by 14–17% relative to the standard ssGBLUP in this analysis. Compatibility statistics support these findings, in which correlations between **G** and **A** increased by 16–38%, regression slopes moved closer to unity, and genomic diagonal and off-diagonal values were better scaled, confirming improved consistency between pedigree and genomic information [38,76]. The altering of **H^−1^** through scaling (τ = 1, ω = 0.60), blending (β = 0.20), and inclusion of UPG contributed to these improvements. These findings align with previous work showing that ω and τ adjustments control the balance between pedigree and genomic information in **H^−1^** [77,78]. The UPGs also contributed to improved predictions, with ssGBLUP_upg alone increasing accuracy by more than 3% and reducing bias by over 11% relative to ssGBLUP. Their inclusion enhanced connectedness across animals with incomplete pedigrees, consistent with earlier studies [43,79]. Despite the greater gains of the adjusted models, suggesting that UPG effects were amplified when combined with scaling and blending, the independent contribution of each adjustment remains to be clarified.

Prediction accuracy and bias are often inversely related, with higher accuracy typically associated with lower bias, as the regression of EBVs from the full dataset on GEBVs from the reduced dataset approaches unity [50,80]. In this study, the ssGBLUP_adjusted0 model showed less bias relative to the standard ssGBLUP. However, the regression coefficients observed in this study deviate from the acceptable range of ±15% from 1 [81], although they remain consistent with previous research [80,82]. Regression coefficients below 1 indicate inflation of GEBVs, meaning that predicted genetic differences among animals are exaggerated relative to realized performance [83]. Such inflation is commonly linked to incompatibility in level and scale between the **A** and **G** matrices, selective genotyping, preferential treatment, and incomplete correction of **A**-**G** matrices discrepancies [24,30,31,32,33,48,49,50]. Therefore, regression coefficients closer to unity under the ssGBLUP_adjusted models reflect improved calibration of GEBVs, confirming the benefits of combining scaling, blending, and UPG inclusion.

Beyond accuracy and bias, the ssGBLUP_adjusted models demonstrated improved calibration of predicted breeding values, with reduced overestimation and tighter dispersion (Figure 3). To further explore calibration patterns, this study evaluated underestimated and overestimated GEBVs within a validation population of 390 animals for fat yield. This analysis highlighted how adjusted_ssGBLUP0 reduces over-dispersion, and the adjusted models also produced a lower mean and narrower GEBVs range (Supplementary Table S3). Although this is a measurement not previously reported, it supports improved calibration and more reliable predictions [36,78]. Therefore, the results of this study confirm that adjustments to the ssGBLUP model strengthen the stability and interpretability of genomic evaluations under the population structure examined in this study.

The improvements observed in this study should be interpreted with consideration of several methodological and population-related limitations. For instance, the validation strategy used reflects realized prediction accuracy under practical breeding conditions, where validation animals without phenotypic records can benefit from phenotypic and genomic information from relatives [72]. However, some young animals may also have contemporaries or relatives with missing phenotypes but available genotypes, which could further influence information flow and prediction accuracy, although this was not explored in this study. In addition, the ssGBLUP models assume a predominantly additive genetic architecture and accurate representation of relationships through **A** and **G** matrices [23,25,26]. Consequently, the performance of the model is sensitive to reference population size, composition, connectedness, selective genotyping, and preferential treatment [44,68,69,70,71,72]. Therefore, using the same scaling, blending, and tuning parameters for all traits and across the population assumes that these parameters are equally optimal in every case, which may not be true. Additionally, the relatively small number of genotyped animals in this study limits the ability to generalize these results to populations with more genotyped animals or different breeding structures.

## 5. Conclusions

The ability of the ssGBLUP_adjusted models to increase prediction accuracy, reduce bias, and improve matrix compatibility for genomic prediction of milk, protein, and fat yields in South African Holstein cattle was highlighted. Incorporating UPG into the ssGBLUP model led to significant improvements in prediction accuracy and bias, emphasizing the potential benefits of increasing genetic diversity in the reference population. Therefore, adjusting the **H^−1^** matrix through blending, tuning, scaling, and inclusion of UPG and subsequently combining them is recommended to enhance the accuracy and reduce bias in genomic predictions in South African Holstein cattle. These adjustments also improved compatibility between the **A** and **G** matrices, resulting in more consistent scaling of genetic information across genotyped and non-genotyped animals. However, the low prediction accuracy and bias observed highlight the need for further work, particularly in expanding genotyping efforts to increase the number of animals with genotypic data in South African Holstein herds. It is recommended that genotyping expansion include young cows, locally used bulls, and animals from underrepresented herds to enhance connectedness and reduce bias, complementing the influence of imported sires. This would be essential for improving prediction accuracy, especially in developing countries with fewer genotyped animals. Future research should continue refining the ssGBLUP model and exploring additional methods for improving genomic predictions in South African dairy cattle.

## Figures and Tables

**Table 1:** Number of records and animals used in this study.

Item	Number
Final pedigree animals	541,325
Unique sires *	9355
Unique dams *	328,929
Phenotypic records	696,413
Cows with phenotypes	354,228
Herds	1991
Herd-year-season	22,410
Genotyped animals	1221
Genotyped bulls	78
Genotyped cows	1143
Genotypes in the full dataset	1221
Genotypes in the reduced dataset	833
Genotypes in the validation dataset	388
SNP markers	41,407

* Unique sires and unique dams indicate animals appearing at least once as a sire or dam in the pedigree and are subsets of the total pedigree animals.

**Table 2:** Parameter parameters for **H^−1^** matrix across ssGBLUP model configurations.

Model	Blending (β)	Scaling (τ, ω)	Tuning	Inbreeding (A^−1^)	UPG
1. ssGBLUP	0.05	(1, 1)	√	✗	✗
2. ssGBLUP_Fx	0.05	(1, 1)	√	√	✗
3. ssGBLUP_upg	0.05	(1, 1)	√	✗	√
4. ssGBLUP_adjusted0	0.20	(1, 0.60)	✗	√	√
5. ssGBLUP_adjusted1	0.20	(1, 0.60)	√	√	√

**ssGBLUP**—single-step genomic best linear unbiased prediction; **A^−1^**—inverse pedigree relationship matrix; **τ**, **ω**—scaling factors; UPG—unknown parent group; √—included; ✗—excluded.

**Table 3:** Summary statistics of relationship matrix elements across models.

Elements	Matrix	Model	Number	Mean	Min	Max
Diagonal	**A_22_**	All	1221	1.012	1.000	1.161
	**G**	ssGBLUP	1221	1.012	0.935	1.255
		ssGBLUP_adjusted0		0.998	0.932	1.200
		ssGBLUP_adjusted1		1.012	0.945	1.215
Off-diagonal	**A_22_**	All	1,489,620	0.011	0.000	0.605
	**G**	ssGBLUP	1,489,620	0.011	−0.104	1.009
		ssGBLUP_adjusted0		0.002	−0.097	0.851
		ssGBLUP_adjusted1		0.011	−0.088	0.864

**Table 4:** Compatibility statistics between **G** and **A**_22_ across ssGBLUP models.

Elements	Model	Correlation	b_0_	b_1_
Diagonal (**G** and **A**)	ssGBLUP	0.54	0.005	0.62
	ssGBLUP_adjusted0	0.64	−0.006	0.68
	ssGBLUP_adjusted1	0.64	0.004	0.68
Off-diagonal (**G** and **A**)	ssGBLUP	0.62	−0.029	0.68
	ssGBLUP_adjusted0	0.71	−0.034	0.73
	ssGBLUP_adjusted1	0.71	−0.024	0.73

b_1_—regression coefficient; b_0_—intercept.

## Data Availability

The datasets generated and/or analyzed during the current study belong to the DGP.

## Supplementary Materials

The following supporting information can be downloaded at: https://www.mdpi.com/article/10.3390/ani16030357/s1, Table S1: Variance components and genetic parameters for milk, protein, and fat yield across BLUP, ssGBLUP, and adjusted ssGBLUP models for Holstein cattle. Table S2: Regression parameters and statistical significance for ssGBLUP and ssGBLUP_adjusted0 models in the 390 validation animals without phenotypes, including intercept (b_0_), slope (b_1_), coefficient of determination (R^2^), *p*-values, and 95% confidence intervals for b_1_. Table S3: Descriptive statistics for GEBVs of 390 validation animals for fat yield (kg) in Holstein cattle, including minimum, maximum, and mean values for ssGBLUP and ssGBLUP_adjusted0 models.
