# Supplementary material for: Evaluating Adjusted ssGBLUP Models for Genomic Prediction and Matrix Compatibility in South African Holstein Cattle
Source: Animals (Basel). 2026 Jan 23;16(3):357. doi: 10.3390/ani16030357 (PMC12897264; doi:10.3390/ani16030357)
Supplement: Supplementary file 1 [file animals-16-00357-s001.zip › animals-4080181-supplementary.pdf]

Supplementary Table S1. Variance components and genetic parameters for milk, protein, and fat yield across BLUP, ssGBLUP, and adjusted ssGBLUP models.

Trait	Model	σ^2_a	σ^2_{pe}	σ^2_e	h^2	r^2
Milk	BLUP	359510	300210	934820	0.278	0.414
	ssGBLUP	363230	298250	933300	0.280	0.415
	ssGBLUP_adjusted0	366120	296770	933300	0.282	0.415
	ssGBLUP_adjusted1	364750	297500	933300	0.281	0.415
Protein	BLUP	351.400	370.520	1317.400	0.211	0.354
	ssGBLUP	354.090	369.100	1315.700	0.212	0.355
	ssGBLUP_adjusted0	357.060	367.510	1315.700	0.213	0.355
	ssGBLUP_adjusted1	356.000	368.090	1315.700	0.213	0.355
Fat	BLUP	289.300	290.640	853.310	0.253	0.405
	ssGBLUP	292.210	289.180	851.740	0.255	0.406
	ssGBLUP_adjusted0	295.160	287.630	851.740	0.257	0.406
	ssGBLUP_adjusted1	293.870	288.320	851.740	0.257	0.406

σ^2_a – additive genetic variance; σ^2_{pe} – permanent environmental variance; σ^2_e – residual variance; h^2 – heritability; and r^2 – repeatability.

Supplementary Table S2 Regression Parameters and Statistical Significance for ssGBLUP and ssGBLUP_adjusted models in each 390 with genotypes and without phenotypes for Holstein Cattle

	Milk		Protein		Fat	
Analysis	ssGBLUP	ssGBLUP_adjusted0	ssGBLUP	ssGBLUP_adjusted0	ssGBLUP	ssGBLUP_adjusted0
b_0	-19.48	-131.36	0.19	-2.03	-2.01	-5.00
b_1	0.30	0.44	0.31	0.47	0.36	0.53
R^2	0.05	0.08	0.08	0.12	0.09	0.13
P value	0.00	0.00	0.00	0.00	0.00	0.00
CI_Lower	0.17	0.30	0.21	0.34	0.24	0.40
CI_Upper	0.42	0.59	0.41	0.59	0.47	0.67

Regression intercept (b_0), slope (b_1), coefficient of determination (R^2), probability that $b_1 \neq 0$ (P value), and 95% confidence interval for b1 (CI Lower and CI Upper)

Supplementary Table S3 Descriptive Statistics for GEBVs of 390 validation animals for Fat Yield (kg) in Holstein Cattle

GEBV	Minimum	Maximum	Mean
ssGBLUP	-11.71	48.94	23.15
ssGBLUP_adjusted0	-3.57	43.31	21.17

Genomic estimated breeding values (GEBVs); and single-step genomic best linear unbiased prediction (ssGBLUP)
